# Questionable role of opioids for analgesia in renal colic and its urological interventions

**DOI:** 10.1002/bco2.70038

**Published:** 2025-06-11

**Authors:** Anna Krieger, Nadim Zaidan, Philip Zhao, James F. Borin, David S. Goldfarb

**Affiliations:** ^1^ Department of Medicine New York University Langone Health New York NY USA; ^2^ Department of Urology New York University Langone Health New York NY USA; ^3^ Kidney Specialists of Minnesota Minneapolis MN USA; ^4^ Department of Medicine Northwell Staten Island University Hospital Staten Island NY USA; ^5^ NYU Grossman School of Medicine New York NY USA; ^6^ Nephrology Division New York University Langone Health and NY Harbor VA Medical Center New York NY USA

**Keywords:** acetaminophen, anti‐inflammatory agents, kidney calculi, non‐steroidal, opiates, opioids

## Abstract

**Objectives:**

To review the different analgesic modalities and benefits of non‐opioid pain management options as well as their evidence‐based, established superiority, compared to opioid medications.

**Materials:**

We review the updated literature about pain management of renal colic, a prevalent and painful urologic condition. Prescribers must know the efficacy, safety and possible ramifications of analgesic selections.

**Results:**

Commonly prescribed medications in the United States (US) include non‐steroidal anti‐inflammatory drugs (NSAIDs), acetaminophen, and opioids. In the context of the current epidemic of death from overdoses of opioids in the US, the frequency of opioid prescribing for renal colic is likely excessive, problematic and potentially remediable. We also present analgesic modalities revolving around interventions with peri‐procedural pain management for ureteroscopy and percutaneous nephrolithotomy. After touching on the implications of misguided opioid use, especially in the context of kidney stone disease, and despite the evidence and consensus guidelines supporting NSAIDs in renal colic, current evidence has shown that many clinicians continue to prescribe opioids as first‐line treatment. Finally, we highlight current efforts targeted at the reduction of opioid use and prescription in the setting of provider education and decision aids in curbing misguided opioid use in renal colic.

**Conclusions:**

While the evidence against treating kidney stones with opioids is clear, more work is needed to shift current practices to reflect that renal colic is a non‐opioid‐requiring condition.

## 1 INTRODUCTION

Nephrolithiasis is a common urologic problem. The recent National Health and Nutrition Examination Survey (NHANES) demonstrated that kidney stones affect 1 in 10 people in the United States (US).^1^ The NHANES also identified a steady percentage increase with time, reporting kidney stone prevalence of 3%, 5% and 10% in 1980, 1994 and 2016, respectively.^2^ Renal colic, the pain resulting from acute obstructing kidney stones, accounts for millions of visits to emergency departments (EDs) and ambulatory clinics annually.^3^ The rate of ED visits for acute renal colic in 2000 was 226 per 100 000 population with a total cumulative cost for caring for patients with urolithiasis estimated at US $2.1 billion in that year (US $971 million inpatient, US $607 million outpatient and US $490 million for emergency services).^4^

Patients typically present with varying degrees of pain, ranging from mild aches to severe discomfort which can be associated with nausea, vomiting, haematuria, urinary frequency and urgency.^5^ Symptoms come on suddenly, prompting many to seek medical attention. Given the frequency and acute presentation of renal colic, it is important for clinicians to be familiar with options for pain control. Furthermore, beyond the prevalence of this condition and the acuteness and pain associated with its presentation, nephrolithiasis is highly recurrent, with up to 2 in 5 patients experiencing at least a second episode after 15 years.^6^

Prescribers must know the efficacy, safety and possible ramifications of analgesic selections. Commonly prescribed medications include non‐steroidal anti‐inflammatory drugs (NSAIDs), acetaminophen (paracetamol) and opioids.^7^ Studies comparing these agents support the use of NSAIDs as first‐line treatment. However, many clinicians in the United States continue to favour opioids. According to the 2016 National Hospital Ambulatory Medical Survey (NHAMCS), 63.7% of ED patients diagnosed with renal calculus were prescribed opioids on discharge.^8^ Similarly, based on a survey conducted among endourologists, approximately 25% of respondents prescribe opioids more than half the time after ureteroscopy.^9^

In the context of the current epidemic of death from overdoses of opioids in the US,^10^ the frequency of opioid prescribing for renal colic is likely excessive, problematic and potentially remediable. In this article, we review pain management modalities for renal colic, as well as peri‐operative analgesia for nephrolithiasis‐related urological interventions. We will explore the implications of misguided opioid use and efforts to limit their use.

## 2 PAIN IN RENAL COLIC

Pain is a common component of renal colic. It is sudden, progressive and radiates anteriorly and downwards into the genitalia. Stone obstruction in the urinary tract increases ureteral peristalsis, wall tension and renal pelvis pressure. As a result, prostaglandins are synthesized and released, inducing vasodilatation and smooth muscle spasm^11^; both further compound intrarenal pressure. Together, these factors result in pain through chemoreceptor activation and stretching of submucosal free nerve endings.^12^ The location of the stone often dictates the sensation of pain. Those with renal pelvis or upper ureteral obstruction may experience flank pain or tenderness, whereas those with lower ureteral obstruction may have pain that radiates to the groin. As many as 80% of stones less than 5 mm will pass spontaneously, relieving the obstruction and immediately resolving pain.^13^ The size and location of a stone often determine the likelihood of passage.^14^ Patients with kidney stones were followed for 20 weeks, and passage rates were found to correlate with stone size (Table 1). Size also determines the time it takes to pass a ureteral stone **(**Table 1 **)**. While waiting for the stone to pass, analgesia is generally required.

**TABLE 1:** Stone passage time and rate by size (Miller & Kane, 1999^15^; Jendeberg et al., 2017^14^).

Stone size (mm)	Passage time (mean number of days)	Passage rate at 20 weeks (%)
< 2	8	98
3	12	98
4	22	81
5		65
6		33
≥ 6.5 mm	‐‐‐	9

## 3 ANALGESIC MODALITIES AND BENEFITS OF NON‐OPIOID PAIN MANAGEMENT

Prompt pain control represents a mainstay of treatment in acute renal colic. Factors affecting analgesic selection include effectiveness, side effects, cost and availability of drugs, as well as patient and provider preferences. A study in 1999 compared the efficacy of opioids and NSAIDs and reported greater pain relief in acute renal colic patients treated with ketorolac compared with meperidine in the ED.^16^ Similar findings were reported in a large 2016 double‐blind RCT in which ED patients presenting with renal colic were assigned to receive intramuscular (IM) diclofenac, intravenous (IV) paracetamol or IV morphine and monitored to assess analgesic effect.^17^ The primary outcome, defined as the proportion of participants achieving ≥ 50% reduction in initial pain score at 30 minutes, occurred in 68%, 66% and 61% of diclofenac, paracetamol and morphine groups, respectively. Compared to morphine, IM diclofenac was significantly more effective at achieving pain control and resulted in greater sustained pain relief at 60 minutes and lesser need for rescue analgesia compared to morphine and paracetamol, reflecting the durability of pain control with diclofenac. Acute adverse events were significantly lower in the diclofenac and paracetamol groups than in the morphine group.^17^

Based on extensive data, NSAIDs, as agents inhibiting prostaglandin synthesis in the setting of acute renal colic, have consistently shown superior evidence of analgesic efficacy compared to antispasmodics or placebo.^12^ Multiple meta‐analyses, of 20 RCTs (1613 patients) in 2004 and subsequently of 65 RCTs (8633 patients) in 2019, found NSAIDs to be superior to opioids, paracetamol and placebo, both in efficacy and safety for acute renal colic.^18^, ^19^ Improvements in visual analogue scale (VAS) pain scores were greater in patients treated with NSAIDs compared with opioids (mean difference 4.60 points on a 100‐point scale), the need for rescue medication was lower (RR 0.75) as was the likelihood of emesis in patients receiving NSAIDs.^19^ NSAIDs were superior in pain variance at 30 minutes, failure of complete relief at 30 minutes and need for rescue analgesia. NSAIDs also had fewer side effects. Opioids resulted in increased vomiting and other nonspecific adverse events.^18^ Interestingly, NSAID effect is not necessarily dose or drug‐specific, with 10‐, 20‐ and 30‐mg doses of IV ketorolac achieving similar efficacy for renal colic analgesia in an ED‐based RCT^20^ and 800 mg of IV ibuprofen, a cheaper alternative, more effective than 30 mg of IV ketorolac, with a significantly higher percentage of complete relief after 60 minutes.^21^ Of note, guidelines published in 2019 by the National Institute of Health and Care Excellence highlight that there are limited data regarding the effectiveness of oral or rectal NSAIDs in comparison to intravenous or intramuscular NSAIDs as assessed in the clinical research literature.^22^ A multidisciplinary review from the Agency for Healthcare Research and Quality (AHRQ) evaluated 183 RCTs related to pain management of conditions as diverse as musculoskeletal, kidney stone, post‐operative, dental, sickle‐cell or neuropathic pain.^23^ Opioids were found to be less effective than acetaminophen for acute kidney stone pain. Indeed, compared with acetaminophen, a single administration of morphine increased the likelihood of persistent pain (pain greater than 2 on a 0 to 10 scale) at 60 minutes and had a similar likelihood of leading to rescue medication use, suggesting that IV acetaminophen is a feasible alternative to IV narcotics for first‐line management of acute renal colic.^23^

While NSAIDs, acetaminophen and opioid use in renal colic have been frequently studied, research on other non‐opioid and non‐pharmacological modalities is increasing. Ketamine has shown benefit with minimal side effects when used in conjunction with NSAIDs.^24^ However, when used alone in comparison to NSAIDs, ketamine offered equal pain relief but higher rates of dizziness, agitation and hypertension.^25^ Lidocaine has also been examined in renal colic, but with conflicting results.^26^ Initial studies on acupuncture have shown positive results,^27^ including an RCT demonstrating greater and faster pain control with acupuncture compared to titrated morphine in ED patients with renal colic.^28^ Aromatherapy as adjunctive therapy has also been examined in the ED setting. The addition of rose essential oil to conventional therapy (i.e. diclofenac) resulted in a significantly greater reduction of pain compared to diclofenac monotherapy in an RCT.^29^

## 4 PROCEDURAL ANALGESIA: UROLOGICAL INTERVENTIONS TREATING NEPHROLITHIASIS AND ITS ASSOCIATED PAIN

Similar to pain management in symptomatic renal colic, surgical procedures addressing nephrolithiasis are increasingly highlighted as a cornerstone of prompt analgesia.^30^ Considerations should however be made regarding the peri‐procedural analgesia required for acute interventions such as ureteroscopy (URS) and percutaneous nephrolithotomy (PCNL).^31^ Across the published literature regarding urological interventions for nephrolithiasis, the last two decades saw an increase in the share of total treatment for urolithiasis by URS, stable use of PCNL and decreased recourse to open surgery and extracorporeal shockwave lithotripsy (ESWL).^32^

Based on the current evidence in the literature, the AUA has been advocating for an opioid‐free (OF) course after URS.^33^ A protocol developed in a pilot study showed that an opioid‐free discharge protocol can effectively decrease opioid prescriptions after URS without affecting postoperative routine follow‐up.^34^ A retrospective analysis of a prospective adult cohort evaluated trends of opioid prescription after URS in 13 143 patients in 28 practices.^35^ Among those not prescribed opioids, there was no associated increased ED utilization after discharge.^35^ Key components of OF URS include thorough patient counselling and transparent discussion of expected pain levels after ureteroscopy/stent. Pain severity post‐URS normalizes to baseline by postoperative day 7 and its interference with daily activities normalized by 14 days, a duration shortened after stent removal.^36^ A meta‐analysis evaluated OF‐discharges after URS and retrograde intrarenal surgery (10 studies, 6786 patients), showing that an OF approach was associated with less ED utilization, highlighting the adequacy of this pain management regimen.^37^ NSAIDs have thus been identified as cornerstones of peri‐procedural analgesia in patients going for URS. Ketorolac at anaesthesia induction for URS was associated with a major decrease in opioid need, representing an independent predictor of decreased peri‐operative narcotic requirement.^38^ Similarly, NSAIDs were non‐inferior to opiates post‐URS with similar physician contact and analgesia and earlier convalescence in comparison to opioid prescription.^39^ In a review of analgesic modalities post‐URS, lower rates of the need for rescue opioids were noted (between 3.7% and 29%) with NSAIDs prescription.^40^ A meta‐analysis of 13 studies and a total of 1408 patients highlights the beneficial effect of antimuscarinics alone and α‐blockers alone in stent‐related discomfort reduction.^41^ That said, opioid requirement after URS remains substantial, with 12% of patients undergoing URS found to require supplemental prescription of opioids within one month in a retrospective cohort.^42^

Analgesia is also required after PCNL, although fewer data are available in the literature regarding pain management for this procedure. The pain associated with PCNL essentially stems from the puncture performed into the kidney's collecting system, with the goal of analgesia to prevent intra‐operative discomfort and opioid use after surgery.^43^ Meta‐analysis suggests that peripheral nerve blocks, with the most extensively studied at the paravertebral, erector spinae and intercostal nerves, may reduce the need for opioids post‐operatively.^44^ Additionally, infiltration of a local anaesthetic around the nephrostomy tract might lead to fewer analgesic requirements after the intervention.^45^ Based on the Enhanced Recovery After Surgery (ERAS) pathway, a peri‐operative multi‐modal approach targeting early recovery after major surgeries, protocols for post‐PCNL analgesia have been evaluated and suggest they could also contribute to less opioid consumption, with an emphasis on NSAID prescription.^46^, ^47^

## 5 IMPLICATIONS OF MISGUIDED OPIOID USE

Initially, overdose mortality during the early phase of the opioid epidemic primarily involved prescription medication, and subsequently, overdoses involved heroin and synthetic opioid consumption.^48^ Between 1999 and 2018, approximately half a million people died from either a prescription or illicit opioid‐induced overdose.^10^ More specifically, by 2017, an opioid was involved in 7 out of 10 deaths secondary to drug overdoses, with 60% of opioid deaths secondary to synthetic drugs.^49^

The risk of opioid dependence and its well‐established morbidity and mortality must be accounted for when selecting narcotics to treat renal colic, especially given the high rates of kidney stone recurrences. Analysis from NHANES showed that a history of kidney stones was independently associated with 27% increased odds of current narcotic use.^50^ The number of stones passed also correlated with increased opioid prevalence, with 0, 1, ≥ 2 prior stones resulting in 6%, 9% and 14% rates of current opioid use, respectively.^50^ Similar associations between kidney stones and narcotics were also found in the Medical Expenditure Panel Survey (MEPS). Data revealed that stone formers were more than five times more likely to have received an opioid prescription compared to those without stones.^51^ Furthermore, MEPS showed that opioid prescriptions for nephrolithiasis often resulted in repeated and prolonged opioid use, with 50% and 22% of stone formers still filling opioid prescriptions at 6 months and 1 year, respectively.^52^ A higher number of kidney stone‐related encounters, evaluated in multiple settings (ER, inpatient and outpatient), was found to increase the odds of subsequent diagnosis of an opioid‐related disorder.^53^ The same association was found after urological procedures, after which opioid prescription was significantly associated with increased use of opioids one year after the intervention.^54^ Analysis of the MarketScan Research Database, a large commercial database, between 2014 and 2017, showed that compared to other opioids, tramadol had a significant association with higher odds of novel persistent use.^55^ Risk factors for opioid use disorder in patients undergoing urological procedures included younger age, inpatient surgery, length of stay and non‐private insurance.^56^ Similarly, after undergoing URS, among 27 740 patients (approximately half of whom never had an opioid prescription before), 6.2% of the opioid‐naïve patients were found to have novel persistent opioid use after the procedure.^57^

Opioid diversion is another serious ramification to consider. A recent national survey found that up to 92% of patients who fill an opioid prescription will have unused medication; 20% will share these leftovers.^58^ Many reported not learning how to safely store or properly dispose of excess pills.^58^ In patients evaluated after surgery in Michigan, only one‐quarter of opioids prescribed were used and notably, the size of prescription was most strongly associated with opioid use, highlighting the need to focus on reported consumption to optimize post‐procedural prescription to adequately meet analgesia requirements.^59^ Within the kidney stone population, half of patients will keep unused narcotics after renal colic episodes.^31^ The implications of leftover opioids and ease of diversion cannot be overlooked. Approximately 75% of prescription opioid misusers obtain the drug from a family member or friend, whether through gifting, buying or stealing.^60^ Doctor “shopping” is another concern after URS, potentially leading to overprescription and higher amounts of circulation opioids in communities.^61^

## 6 TREATMENT CONSENSUS FOR KIDNEY STONE PAIN – WHAT THE GUIDELINES SAY FOR NEPHROLITHIASIS ANALGESIA

Evidence‐based guidelines exist for pain management of renal colic. Aligning with the above findings, the last couple of European Association of Urology (EAU) guidelines on urolithiasis outline the superiority of NSAIDs in renal colic and strongly recommend selecting NSAIDs or paracetamol as first‐line pharmacotherapy **(**Tables 2, 3 **).** ^10^, ^30^, ^31^, ^32^, ^33^, ^34^, ^35^, ^36^, ^37^, ^38^, ^39^, ^40^, ^41^, ^42^, ^43^, ^44^, ^45^, ^46^, ^47^, ^48^, ^49^, ^50^, ^51^, ^52^, ^53^, ^54^, ^55^, ^56^, ^57^, ^58^, ^59^, ^60^, ^61^, ^62^ Offering opioids was deemed a weak recommendation. EAU guidelines also include analgesic recommendations for outpatient management of recurrent pain, writing, “for patients with ureteral stones that are expected to pass spontaneously, NSAID tablets or suppositories (e.g. diclofenac sodium, 100‐150 mg/day, 3‐10 days) may help reduce inflammation and the risk of recurrent pain”.^62^ Similarly, British guidelines explicitly stress that opioids can be considered if both NSAIDs and IV paracetamol are found to be ineffective or contraindicated.^22^ The latest guidelines of the EAU have also emphasized the role of early urological intervention in pain relief.^30^

**TABLE 2:** EAU guidelines on urolithiasis: summary of evidence for management of renal colic.

Guidelines	2020	2024
Summary of evidence	Non‐steroidal anti‐inflammatory drugs are very effective in treating renal colic and are superior to opioids	Non‐steroidal anti‐inflammatory drugs are very effective in treating renal colic and are superior to opioids. For symptomatic ureteral stones, stone removal as first‐line treatment is a feasible option in selected patients.
Level of evidence	1b	1b

**TABLE 3:** EAU 2020 & 2024 guidelines on urolithiasis: recommendations for management of renal colic.

Recommendation strength rating	2020	2024
Strong	Offer a non‐steroidal anti‐inflammatory as the first drug of choice; e.g. metamizol*** (dipyrone); alternatively paracetamol or, depending on cardiovascular risk factors, diclofenac*, indomethacin or ibuprofen**.	Offer a non‐steroidal anti‐inflammatory as the first drug of choice; depending on cardiovascular risk factors and side effects. Offer renal decompression or uereteroscopic stone removal in case of analgesic refractory colic pain
Weak	Offer opiates (hydromorphine, pentazocine or tramadol) as a second choice.	Offer opiates (hydromorphine, pentazocine or tramadol) as a second choice.

* Affects glomerular filtration rate (GFR) in patients with reduced renal function.

** Recommended to counteract recurrent pain after ureteral colic.

*** Maximum single oral dose recommended 1000 mg, total daily dose up to 5000 mg, not recommended in the last three months of pregnancy.

From Table 3.4.1.1. Summary of evidence and recommendations for the management of renal colic 2024.

In contrast to the EAU, the American Urologic Association (AUA) currently does not specifically address pain management in their urolithiasis guidelines, which were last reviewed in 2019. As outlined in its methodology, “Studies addressing acute pain management and treatment to promote expulsion of stones were excluded”.^63^ While not included in its guidelines, the AUA does give information regarding pain management of kidney stones in its medical school curriculum. It writes, “Since most stone patients present with pain, analgesia must also be addressed. Narcotics and nonsteroidal anti‐inflammatory drugs (NSAIDs) are commonly used for pain relief. In most randomized, blinded studies of NSAIDs versus narcotics, NSAIDs have shown equal or greater efficacy of pain relief and a shorter time to reach adequate analgesia with equal or fewer side effects”.^64^ Lastly, based on the opioid prescription guidelines published by the CDC in 2016,^65^ the AUA encourages opioid discontinuation in its *Choosing Wisely* campaign listing 15 Fifteen Things Physicians and Patients Should Question.^66^ The 2022 updates reiterate “Don't continue opioid analgesia beyond the immediate postoperative period; prescribe the lowest effective dose and number of doses required to address the expected pain”.^66^

## 7 COMMON PRACTICES

Despite the evidence and consensus guidelines supporting NSAIDs in renal colic, many clinicians continue to prescribe opioids as first‐line treatment. On a national level, this practice results in thousands of new narcotic prescriptions written annually. According to the 2016 NHAMCS, narcotics were prescribed for more than 300 000 ED visits for patients diagnosed with renal calculus.^8^ Portis et al. analysed analgesic selection for renal colic in a Midwestern US metropolitan health system, with most patients receiving opioids (79%), many of whom were prescribed only opioids (40%).^67^ Similar findings were noted when surveying opioid use in an Eastern US urban hospital, where 43% of patients with kidney stones were treated with narcotics; 23% were treated with narcotics alone.^68^ Upon discharge, 69% of these patients received an opioid prescription.^68^ In a retrospective analysis of a large cohort of 22 609 veterans with nephrolithiasis between 1999 and 2014, kidney stones were surgically treated in 1976 cases, with 80.1% of these patients being prescribed opioids.^69^ While the type of surgery did not affect the doses prescribed, a PTSD diagnosis was associated with significantly higher opioid doses.^69^

Clinical practice regarding opiate use differs when comparing North America and Europe. In a Canadian study, between 2013 and 2017, two‐thirds of the 101 896 opioid‐naïve patients seen for urolithiasis were prescribed opioids (9% of whom exhibited persistent opioid use more than 90 days after their initial visit).^70^ Bounces et al. analysed prescribing habits for renal colic in US and French emergency settings.^71^ Opioids were more commonly used by US physicians compared to French physicians (64% and 38%, respectively). There was also a discrepancy seen in NSAID use (68% and 88% in the US and France, respectively). Of note, analgesia results were similar between the two countries despite differing analgesic selections.^71^

## 8 EFFORTS TO CURB OPIOID USE

Since the Department of Health and Human Services declared the opioid crisis in 2017, patterns of prescription of opioids for urolithiasis in ED were analysed during a 5‐year period, pre‐declaration (2014–2016) and post‐declaration (2017–2018).^72^ While opioid prescription occurred in 41.1% of cases (211 out of 513 million ED visits during the whole study period), there was a 4.3% not‐statistically significant decrease in opioid prescription between the 2 study periods.^72^

On a practical level, to address the harms associated with narcotics in nephrolithiasis, various institutions have implemented quality improvement efforts to reduce opioid use in the management of renal colic. A retrospective analysis of urolithiasis encounters receiving analgesia before and after implementation of a multi‐modal pain management guideline showed a decreased opioid prescription in the ED after the protocol took effect but noticed higher rates of inpatient admissions.^73^ A programme in which ED physicians received structured teaching on the effectiveness of acetaminophen and ketorolac in renal colic encourages providers to select such non‐narcotic analgesic options.^67^ Following the commencement of this initiative, the selection of opioid‐sparing agents as first‐line treatment of renal colic increased (36% to 51%). Overall administration of acetaminophen increased (1% to 26%), while opioid use decreased (79% to 71%). Patients presenting to the ED post‐teaching were less likely to receive narcotics when adjusting for age, sex, stone size, location and initial pain score.^67^ In a pre‐post study, the implementation of an ERAS protocol after URS (28 patients pre‐ERAS and 52 post‐ERAS) led to a significant decrease in opioid prescriptions.^74^ These effects may be modest when considering the relatively high frequency of opioid prescriptions.

Broader opioid reduction strategies have also worked to decrease narcotic use in nephrolithiasis. An ED initiative that educated providers on pharmacologic options for acute pain management also promoted an analgesic approach that targeted the pathways involved in specific pain syndromes, including renal colic. This framework was then applied to a complaint‐based, non‐opioid medication selection tool made available to prescribers at the time of encounter.^75^ The investigators later analysed the effects of this initiative on pain management in patients presenting with renal colic and found that it decreased opioid utilization in the ED and at discharge by 13% and 26%, respectively.^68^ Finally, these measures are particularly relevant in paediatric and young adult populations, especially given the increased incidence of kidney stones in these populations.^76^ Indeed, among patients 25 years of age and younger seen in the ED for renal colic, 56% were prescribed opioids, a number that was associated with increased odds of opioid use 6 months after the encounter.^77^ In a retrospective review of paediatric urolithiasis patients presenting to a quaternary centre's ED between 2011 to 2022, implementation of a urolithiasis management protocol in 2017 led to a statistically significant reduction in opioid prescription (44% vs 26.7%).^78^

Taken together, the above studies highlight the benefit of provider education and decision aids in curbing misguided opioid use in renal colic; however, it is important to note that these initiatives require time and effort on the part of practitioners, which may limit their feasibility. Fortunately, relatively simple and less time‐intensive interventions can help as well. Another study by Portis et al. shared a protocol in which ED physicians were asked to give time for ketorolac and acetaminophen to take effect prior to administering narcotics for renal colic. As a result, initial therapy with non‐opioid analgesics increased by 250% and usage of narcotics at any time in the ED decreased by 34%.^79^

## 9 CONCLUSIONS, UNANSWERED QUESTIONS AND RESEARCH NEEDS

Renal colic is a frequent problem with extensively studied treatment options. NSAIDs have consistently proven their effectiveness as first‐line analgesic therapy in renal colic, with no study clearly suggesting the superiority of opioids (Table 4). Increasing literature demonstrates the risk of narcotic dependence in patients with kidney stones. It is imperative that providers consider this fact when selecting medications for renal colic, especially given that stones can take weeks to pass and often recur. Despite this, many providers continue to treat kidney stones with narcotics. Furthermore, consensus guidelines regarding an evidenced‐based approach to renal colic analgesia are limited. While the EAU explicitly recommends NSAIDs and paracetamol over opioids, the AUA does not currently specify preferred therapy. We believe the AUA should and will amend their guidelines to include recommendations regarding pain management, emphasizing the benefits of NSAIDs over opioids.

**TABLE 4:** Highlighted studies evaluating analgesia for renal colic.

Study	Design	Intervention	Measures	Findings
Gu et al., 2019	Meta‐analysis; 65 RCTs (n = 8633)	NSAIDs versus opioids versus paracetamol versus combination therapy versus placebo	Pain variance Duration: 30 minutes	IV and IM NSAIDs are superior to opioids, paracetamol and placebo
Pathan et al., 2016	RCT, double‐blind, multigroup (n = 1644)**	IM diclofenac 75 mg/ml versus IV morphine 0.1 mg/kg versus IV paracetamol 1 g/100 ml	NRS Duration: 30 minutes	Compared to morphine, IM diclofenac was significantly more effective at achieving pain control (OR 1.35, 95% CI 1.05–1.73, p = 0.0187). No difference in the effectiveness of morphine compared with paracetamol
Metry et al., 2019	RCT, double‐blind (n = 120)**	IV lornoxicam 8 mg + IV ketamine 0.15 mg/kg versus IV pethidine 50 mg	VAS Duration: 15, 30, 45 and 60 minutes	IV lornoxicam + ketamine resulted in significantly better pain control, lesser need for rescue analgesia and fewer side effects compared to pethidine
Sotoodehnia et al., 2019	RCT, double‐blind (n = 126)**	IV ketamine 0.6 mg/kg versus IV ketorolac 30 mg	NRS Duration: 5, 15, 30, 60 and 120 minutes	Low‐dose ketamine provided equivalent analgesia compared to ketorolac but resulted in higher rates of side effects
Motov et al., 2020	RCT, double‐blind (n = 150)**	Combination IV lidocaine (1.5 mg/kg) + ketorolac (30 mg) versus IV ketorolac 30 mg versus IV lidocaine 1.5 mg/kg	NRS Duration: 30 minutes	Combination IV lidocaine/ketorolac resulted in better pain control compared to lidocaine monotherapy but did not offer benefits over ketorolac alone
Kaynar et al., 2015	RCT (n = 121)**	IV acetaminophen 1 g/100 ml versus IM diclofenac 75 mg versus acupuncture	VAS, VRS Duration: 10, 30, 60 and 120 minutes	All modalities were effective in attenuating renal colic pain. Acupuncture is an analgesic option to consider in patients with contraindications to NSAIDs and/or acetaminophen
Beltaief et al., 2018	RCT (n = 115)**	IV titrated‐morphine (0.1 mg/kg morphine every 5 minutes until pain score dropped by at least 50%) versus 30 minute acupuncture session	VAS Duration: 30 minutes	Acupuncture resulted in significantly greater and faster analgesia compared to morphine
Ayan et al., 2013	RCT, double‐blind, placebo‐controlled (n = 80)**	Conventional therapy (IM diclofenac 75 mg) + aromatherapy (rose essential oil) versus conventional therapy + placebo	VAS Duration: 10 and 30 minutes	Pain scores were significantly lower in the combined conventional therapy + aromatherapy group at 10 and 30 minutes

IV, intravenous; IM, intramuscular; NRS, numerical rating scale; RCT, randomized control trial; VAS, visual analogue scale; VRS, verbal rating scale.

** Emergency department‐based study of adult patients presenting with symptoms consistent with renal colic.

The evidence against treating kidney stones with opioids is clear. Subjecting patients to extended or recurrent opioids not only lacks provision of pain benefits compared to alternative agents, but it also comes with the potential harm of opioid addiction and prescription diversion, affecting both the individual and community at large. NSAIDs and acetaminophen should represent the mainstays of analgesic treatment. More work is needed to shift current practices to reflect that renal colic is a non‐opioid‐requiring condition. While it is promising that institutions are working to reduce misguided opioid use, more studies are needed on the implementation of effective protocols. All the example initiatives described above helped decrease narcotic usage, but the extent of reduction highlights the need for further improvement.

There is also a need to address clinical settings outside of the ED given that renal colic is managed by various types of providers. Ideally, pain episodes should be manageable outside of the ED; strategies devised to achieve less opioid use in other settings have not yet been explicitly developed and implemented. Concurrently, future studies should evaluate the relative benefit of non‐opioid, non‐NSAID analgesics (e.g. lidocaine, medical marijuana) and/or nonpharmacologic interventions (e.g. relaxation, acupuncture) in renal colic. We question whether the transient and reversible effect of NSAIDs to reduce glomerular filtration rates, in patients for whom relief of obstruction will soon lower serum creatinine concentrations, contributes to misguided opioid use. Finally, the prevention of nephrolithiasis should be emphasized, especially given the current trend of rising prevalence. With less stone burden, there is less risk of misguided opioid use and its associated harms.

## AUTHOR CONTRIBUTIONS

AK and DSG conceived the study. AK, NZ, PZ, JFB and DSG reviewed the relevant literature. AK and NZ wrote the first draft. PZ, JFB and DSG edited the manuscript. All authors approved the final version of the manuscript.

## CONFLICT OF INTEREST STATEMENT

The authors declare no conflict of interest.

## INFORMED CONSENT STATEMENT

Not applicable.

## Data Availability

Not applicable.
